# Supplementary material for: A Closer Look at Benchmarking Self-supervised Pre-training with Image Classification
Source: Int J Comput Vis. 2025 Apr 27;133(8):5013–25. doi: 10.1007/s11263-025-02402-w (PMC12289721; doi:10.1007/s11263-025-02402-w)
Supplement: Supplementary file 1 — (pdf 539 KB) [file 11263_2025_2402_MOESM1_ESM.pdf]

Supplementary Materials for
A Closer Look at Benchmarking Self-Supervised Pre-training with Image Classification

A Protocol Survey

Table S.1 shows the survey on classification-based SSL evaluation protocols we conducted at the beginning of this study. We decided to include the most protocols and exclude rarely used ones, i.e., partial fine-tuning and probing with a Support Vector Machine. Note that many papers additionally evaluate task transfer learning (e.g., semantic segmentation, object detection, image retrieval, or video classification), which is not covered in this survey.

Table S.1: Survey on classification-based evaluation protocols for self-supervised learning in a selection of papers. A ✓denotes that the protocol was used in the mentioned paper. knn: k-nearest neighbors probing, LP: linear probing, FT: fine-tuning, FSFT: few-shot fine-tuning (1% or 10%), PFT: partial fine-tuning, SVM: SVM probing, CM: clustering metrics. Note that many papers additionally evaluate task transfer learning (typically on object detection or semantic segmentation tasks) which is not covered in this table.

		In domain						Out of domain		
Method	Year	knn	LP	FT	FSFT	PFT	SVM	knn	LP	FT
Jigsaw [26]	2016			✓						✓
npid [33]	2018	✓		✓	✓		✓			
PIRL [24]	2019		✓	✓	✓				✓	✓
BYOL [18]	2020		✓		✓				✓	✓
SimCLR [8]	2020		✓	✓	✓				✓	✓
MoCo v2 [12]	2020		✓							
ImageGPT [7]	2020		✓	✓					✓	✓
SwAV [6]	2020	✓		✓	✓		✓		✓	
SimSiam [10]	2020	✓	✓							
DINO [5]	2021	✓	✓	✓	✓				✓	
MAE [19]	2021		✓	✓		✓				✓
BEiT [3]	2021		✓	✓						✓
MoCo v3 [11]	2021	✓	✓	✓						
SimMIM [34]	2021		✓	✓						
iBOT [37]	2021	✓	✓	✓	✓					✓
MaskFeat [31]	2021			✓						
SEER [17]	2021		✓	✓	✓				✓	✓
Barlow Twins [36]	2021		✓		✓				✓	
data2vec [2]	2022			✓						
MILAN [20]	2022		✓	✓						
PeCo [14]	2023		✓	✓						
PixMIM [23]	2023		✓	✓	✓					
CAE [9]	2024		✓	✓						✓

B Full Result Tables

Table S.2: *Top-1* classification accuracies (in %). LP: Linear Probing, FT: End-to-end Fine-tuning, FT 10%/1%: Few-Shot Fine-tuning on 10%/1% of the training data, kNN: k-nearest neighbors probing. The superscript "BN" denotes the use of a final batch norm layer. The superscript "N" is the equivalent for k-nearest neighbors probing where the embedding is normalization on training split statistics.

		ImageNet									PascalVOC				
Method	Backbone	kNN	kNN^{N}	LP	LP^{BN}	FT	FT10%	$FT10\%^{BN}$	FT1%	$FT1\%^{BN}$	kNN	kNN^{N}	LP	LP^{BN}	FT
Jigsaw [26]	RN-50	12.0	12.4	28.6	32.5	72.2	50.9	53.8	17.0	22.2	39.0	39.1	44.7	53.7	64.6
rotnet [16]	RN-50	18.5	18.1	35.3	41.0	72.4	52.7	55.2	20.1	24.4	42.0	44.0	53.2	59.0	65.0
npid [33]	RN-50	36.7	37.1	31.5	51.0	71.7	50.5	56.2	10.2	29.9	55.4	54.9	32.5	67.7	59.8
Sela-v2 [1]	RN-50	60.4	61.2	70.4	70.3	75.9	68.4	68.6	50.5	55.5	73.3	68.7	82.1	83.4	87.9
npid++ [24, 33]	RN-50	36.8	38.4	54.0	52.5	72.7	56.7	58.7	27.4	32.8	55.4	54.5	70.5	68.8	74.7
PIRL [24]	RN-50	49.0	49.5	58.1	58.7	73.8	59.9	60.9	34.3	39.5	64.5	61.3	75.1	73.0	79.1
clusterfit [35]	RN-50	35.0	35.7	48.6	49.8	72.0	54.3	56.2	25.2	30.3	54.0	50.2	66.2	66.4	70.1
Deepcluster-v2 [4, 6]	RN-50	64.8	65.7	74.7	73.5	76.5	69.6	70.0	50.0	57.4	75.1	75.4	85.7	85.4	87.5
SwAV [6]	RN-50	63.2	64.3	74.1	73.2	76.3	69.0	70.3	46.0	56.7	73.9	74.2	84.3	84.9	86.5
SimCLR [8]	RN-50	58.1	57.2	66.9	68.1	74.6	65.8	66.6	47.6	53.6	73.1	64.2	81.4	81.9	87.2
MoCo v2 [12]	RN-50	59.4	58.7	60.0	70.4	75.0	61.6	63.9	27.2	48.9	78.3	75.9	67.0	84.2	79.5
SimSiam [10]	RN-50	57.5	57.9	38.1	69.2	74.3	53.7	61.7	8.5	40.2	76.0	72.5	16.0	83.0	54.8
BYOL [18]	RN-50	63.2	64.0	72.7	72.1	75.6	68.2	68.4	50.0	56.3	75.6	74.3	84.3	83.8	86.6
Barlow Twins [36]	RN-50	61.8	62.5	71.5	70.8	74.2	66.2	65.2	47.4	51.4	76.1	73.7	83.8	83.2	84.6
DenseCL [30]	RN-50	48.2	48.8	49.3	63.1	74.5	59.3	61.9	26.8	42.7	75.6	73.2	73.2	80.5	79.7
DINO [5]	RN-50	64.1	64.2	74.4	73.3	76.0	68.4	69.5	45.2	55.5	75.7	74.4	83.5	82.8	86.2
MoCo v3 [11]	RN-50	66.2	66.4	74.5	73.4	76.2	69.4	70.3	51.3	59.1	76.7	73.8	86.1	84.8	87.2
DINO [5]	ViT-B/16	74.7	74.9	76.7	77.6	81.3	75.4	76.6	65.6	69.2	86.5	86.5	87.6	88.6	87.7
iBOT [37]	ViT-B/16	75.8	76.0	78.2	78.9	82.5	77.7	78.7	68.8	71.8	87.0	86.8	88.1	89.7	89.5
MoCo v3 [11]	ViT-B/16	70.3	70.9	73.9	76.3	80.8	74.4	74.8	34.6	66.0	81.8	82.5	80.9	88.3	81.9
MAE [19]	ViT-B/16	26.6	47.7	58.7	66.7	79.3	68.0	69.7	42.4	54.2	34.5	63.2	50.5	80.3	86.4
MaskFeat [31]	ViT-B/16	13.7	30.0	3.7	51.7	76.4	56.5	64.8	7.7	43.7	34.6	51.8	16.0	67.0	55.2
BEiT v2 [29]	ViT-B/16	69.1	70.0	78.4	78.2	84.8	80.5	80.7	68.2	72.8	82.1	82.3	89.1	88.7	91.8
MILAN [20]	ViT-B/16	72.9	73.2	76.4	78.8	84.4	78.9	79.0	67.3	69.4	87.3	87.0	89.0	90.0	91.9
EVA [15]	ViT-B/16	47.1	50.9	60.5	68.7	82.1	71.6	72.9	41.1	52.5	61.0	65.5	47.5	79.3	83.4
PixMIM [23]	ViT-B/16	39.4	49.5	59.3	66.5	79.2	63.3	66.1	25.9	44.3	51.9	65.3	50.4	81.5	80.0

		Cifar100					Caltech-256					CUB				
Method	Backbone	kNN	kNN^{N}	LP	LP^{BN}	FT	kNN	kNN^{N}	LP	LP^{BN}	FT	kNN	kNN^{N}	LP	LP^{BN}	FT
Jigsaw [26]	RN-50	26.7	27.6	27.4	36.7	77.5	26.7	27.3	29.2	42.4	74.0	3.4	4.1	5.1	7.8	52.6
rotnet [16]	RN-50	38.5	38.4	37.5	46.9	79.6	34.5	34.9	41.5	55.6	74.9	5.2	5.3	7.6	13.3	60.1
npid [33]	RN-50	40.4	39.9	23.3	56.5	78.6	46.5	46.3	16.0	67.8	68.5	15.9	15.7	10.7	29.0	32.3
Sela-v2 [1]	RN-50	59.2	57.6	37.9	46.5	85.6	68.6	68.0	74.2	80.7	87.7	17.1	19.7	21.1	41.7	68.4
npid++ [24, 33]	RN-50	39.0	38.5	43.6	54.6	82.4	43.5	43.0	63.9	67.4	80.3	14.4	15.0	22.2	30.1	71.7
PIRL [24]	RN-50	47.7	45.3	35.8	49.2	83.4	57.4	58.2	69.9	72.7	83.2	16.2	16.4	21.5	29.3	71.3
clusterfit [35]	RN-50	49.4	48.9	62.0	62.2	81.0	42.6	48.6	66.6	66.2	79.0	7.2	10.3	18.0	19.3	58.4
Deepcluster-v2 [4, 6]	RN-50	63.1	63.0	45.6	48.6	85.9	73.2	73.7	83.1	86.1	89.2	27.1	33.7	39.0	56.5	73.0
SwAV [6]	RN-50	62.2	62.7	43.8	47.4	85.5	72.0	72.7	80.7	84.7	88.3	24.1	30.3	30.6	52.9	71.4
SimCLR [8]	RN-50	55.6	51.9	33.5	50.9	85.1	64.5	64.0	70.4	79.9	86.1	18.5	19.1	18.9	37.1	63.2
MoCo v2 [12]	RN-50	58.7	57.3	29.3	66.5	83.9	70.4	70.2	39.0	84.0	83.9	20.4	23.4	13.0	45.6	55.3
SimSiam [10]	RN-50	61.4	58.3	20.4	61.2	79.3	69.7	68.1	7.8	83.9	70.9	22.4	24.6	9.6	48.6	26.4
BYOL [18]	RN-50	62.7	59.4	54.2	60.5	85.8	71.0	69.5	81.8	85.3	87.5	35.3	39.0	40.2	58.2	73.4
Barlow Twins [36]	RN-50	61.6	58.4	57.1	57.7	84.1	73.0	71.6	81.9	83.4	86.0	30.4	36.2	42.2	56.0	61.2
DenseCL [30]	RN-50	47.4	49.8	27.1	56.5	83.7	62.0	62.5	49.6	77.0	82.6	14.9	16.9	10.4	30.9	58.8
DINO [5]	RN-50	58.0	56.5	46.0	49.5	85.3	73.1	71.7	81.5	85.0	88.3	31.3	34.8	32.0	53.0	71.6
MoCo v3 [11]	RN-50	64.4	59.2	58.9	61.5	86.2	73.0	68.8	85.2	86.3	88.5	43.9	41.9	49.0	60.4	76.2
DINO [5]	ViT-B/16	76.5	77.0	82.6	83.0	90.6	85.7	86.0	90.6	91.2	92.4	58.6	59.6	78.2	79.2	83.2
iBOT [37]	ViT-B/16	76.7	76.9	82.8	83.2	92.2	86.5	87.0	91.3	91.8	93.4	57.4	59.0	78.2	78.8	84.1
MoCo v3 [11]	ViT-B/16	75.2	75.9	76.5	83.3	88.3	79.6	80.5	79.5	89.5	87.7	43.4	45.3	39.4	71.8	59.0
MAE [19]	ViT-B/16	22.3	48.7	42.2	66.7	85.3	28.7	60.0	34.6	80.5	86.6	5.5	16.5	7.1	44.5	70.2
MaskFeat [31]	ViT-B/16	29.8	44.2	9.6	58.7	84.2	21.8	33.7	6.4	60.1	76.1	4.0	5.6	1.5	15.6	29.5
BEiT v2 [29]	ViT-B/16	74.0	74.4	83.0	83.0	92.3	79.0	79.7	90.2	90.5	94.7	19.5	20.7	50.7	52.9	84.7
MILAN [20]	ViT-B/16	70.6	71.2	70.9	79.7	90.0	87.7	87.8	88.2	92.4	94.5	56.8	57.7	52.0	75.4	83.1
EVA [15]	ViT-B/16	55.2	60.1	54.6	76.6	87.4	53.9	59.5	29.8	82.8	89.0	11.6	13.8	9.0	39.1	65.3
PixMIM [23]	ViT-B/16	43.4	57.5	50.0	71.8	81.1	47.6	62.3	35.8	81.1	81.5	9.6	15.5	8.0	40.8	48.2

ImageNet-D

		Clipart					Infograph					Painting				
Method	Backbone	kNN	kNN^{N}	LP	LP^{BN}	FT	kNN	kNN^{N}	LP	LP^{BN}	FT	kNN	kNN^{N}	LP	LP^{BN}	FT
Jigsaw [26]	RN-50	16.0	16.7	22.1	35.7	65.0	8.8	8.7	9.8	13.4	31.3	15.0	15.1	23.1	32.1	57.5
rotnet [16]	RN-50	23.7	24.7	34.5	46.9	68.2	9.0	10.0	12.9	17.8	34.6	21.7	21.7	34.0	41.9	60.8
npid [33]	RN-50	29.1	28.4	10.6	47.3	65.3	14.0	14.2	5.3	19.1	31.4	30.7	31.0	16.8	45.2	57.4
Sela-v2 [1]	RN-50	35.4	34.3	48.8	55.2	74.1	17.8	18.9	22.8	25.7	42.4	45.3	45.9	56.6	59.1	70.2
npid++ [24, 33]	RN-50	23.5	22.1	40.8	45.5	69.3	13.8	13.8	17.2	18.5	35.2	29.2	29.2	43.6	43.5	62.0
PIRL [24]	RN-50	34.9	34.0	47.8	55.4	71.4	16.4	17.7	20.5	23.9	37.5	39.7	39.8	50.1	52.6	64.7
clusterfit [35]	RN-50	34.6	35.4	52.0	52.5	68.7	14.5	15.3	20.0	20.4	33.6	35.0	36.1	46.4	47.1	61.1
Deepcluster-v2 [4, 6]	RN-50	40.0	40.6	59.8	60.3	74.7	21.1	22.3	28.7	28.9	42.1	49.2	50.3	64.6	63.5	70.7
SwAV [6]	RN-50	38.3	39.1	57.5	59.6	74.4	20.1	21.8	27.3	28.4	42.0	48.1	49.7	63.0	62.9	70.4
SimCLR [8]	RN-50	33.4	34.6	38.6	55.9	73.8	17.2	18.6	16.2	24.9	40.1	44.1	44.3	51.2	57.9	68.6
MoCo v2 [12]	RN-50	36.6	38.9	18.7	62.5	71.5	18.1	20.3	8.1	29.7	37.2	49.0	49.2	33.4	62.0	65.1
SimSiam [10]	RN-50	39.2	41.3	1.0	63.9	67.3	18.8	20.8	1.6	29.6	32.7	48.8	49.0	1.5	63.0	60.5
BYOL [18]	RN-50	40.2	42.2	58.2	63.5	75.2	19.1	21.6	25.3	28.9	42.2	49.8	49.8	62.5	63.7	70.4
Barlow Twins [36]	RN-50	40.4	41.1	62.9	63.0	71.3	20.1	21.1	28.4	28.1	36.7	49.9	49.6	63.7	62.1	67.6
DenseCL [30]	RN-50	31.1	31.9	21.0	54.5	71.4	15.2	15.8	10.3	24.1	36.5	41.8	42.6	33.8	55.4	65.1
DINO [5]	RN-50	37.9	38.8	60.3	61.2	73.6	20.3	22.2	28.6	28.5	41.8	48.9	49.1	64.3	63.3	70.1
MoCo v3 [11]	RN-50	46.8	45.6	64.9	65.4	76.0	21.2	22.3	29.4	30.4	43.1	53.2	51.7	65.7	64.9	71.5
DINO [5]	ViT-B/16	53.4	53.9	67.7	68.1	77.8	29.6	29.8	37.2	37.9	45.4	63.1	63.3	67.3	68.5	73.7
iBOT [37]	ViT-B/16	55.2	55.1	69.5	70.0	80.2	30.6	30.2	38.3	39.1	47.8	63.6	63.7	68.5	69.7	75.9
MoCo v3 [11]	ViT-B/16	50.5	51.0	39.2	68.1	74.0	26.1	26.2	19.6	35.2	40.8	57.7	58.4	53.9	66.4	72.4
MAE [19]	ViT-B/16	21.1	41.4	25.9	61.7	73.4	10.4	18.8	11.2	28.4	40.4	15.6	39.9	32.0	57.7	69.5
MaskFeat [31]	ViT-B/16	14.3	19.8	2.2	43.0	63.1	7.5	10.7	2.0	16.5	26.7	12.0	24.2	3.3	43.8	58.9
BEiT v2 [29]	ViT-B/16	57.8	57.9	72.8	72.1	83.3	26.1	25.8	39.2	38.9	51.7	59.8	60.0	70.5	69.8	79.6
MILAN [20]	ViT-B/16	67.5	68.1	66.5	75.2	82.8	36.4	36.7	34.7	44.8	53.4	65.7	66.0	67.5	72.8	79.8
EVA [15]	ViT-B/16	36.9	40.9	28.8	62.9	74.6	18.8	20.3	12.8	31.0	43.0	37.4	41.5	35.4	59.3	72.5
PixMIM [23]	ViT-B/16	29.6	42.1	26.9	62.1	68.0	14.9	19.5	11.9	28.7	36.6	28.8	42.8	34.5	58.2	66.2

		Quickdraw					Real					Sketch				
Method	Backbone	kNN	kNN^{N}	LP	LP^{BN}	FT	kNN	kNN^{N}	LP	LP^{BN}	FT	kNN	kNN^{N}	LP	LP^{BN}	FT
Jigsaw [26]	RN-50	30.9	32.5	16.4	26.5	71.7	29.2	29.4	43.0	53.0	76.2	14.5	15.1	17.7	27.1	59.1
rotnet [16]	RN-50	35.4	35.0	35.2	49.7	72.0	36.3	36.5	52.2	60.3	77.4	20.5	21.4	29.3	40.4	62.2
npid [33]	RN-50	40.6	37.4	8.4	45.1	70.7	50.5	50.8	36.6	64.3	76.1	25.9	25.8	9.6	38.5	59.3
Sela-v2 [1]	RN-50	32.1	27.2	33.2	42.8	70.5	66.9	67.5	75.3	75.9	82.3	32.1	32.4	44.0	48.7	67.7
npid++ [24, 33]	RN-50	26.4	19.7	29.4	43.3	71.6	49.7	49.9	65.8	64.6	78.4	18.9	18.7	29.1	34.0	63.3
PIRL [24]	RN-50	33.0	24.6	32.2	39.8	71.5	59.5	59.5	69.2	70.9	79.6	32.0	31.2	40.9	46.7	64.6
clusterfit [35]	RN-50	41.1	41.2	51.1	51.4	69.9	52.7	53.0	65.6	66.5	78.2	30.0	30.8	43.2	43.7	61.6
Deepcluster-v2 [4, 6]	RN-50	33.7	34.4	44.1	47.4	71.0	70.8	71.3	80.3	78.7	82.8	35.9	36.5	52.3	53.3	68.4
SwAV [6]	RN-50	32.6	31.4	45.5	50.5	71.3	70.2	70.8	79.3	78.1	82.5	34.7	35.8	50.8	53.3	68.4
SimCLR [8]	RN-50	27.6	18.0	26.1	46.4	71.1	64.7	64.0	70.1	75.0	81.3	32.1	32.2	36.3	49.9	66.7
MoCo v2 [12]	RN-50	33.1	30.8	13.2	47.8	72.2	66.5	66.8	59.3	78.0	80.3	35.9	36.9	17.6	55.9	65.2
SimSiam [10]	RN-50	38.8	35.4	11.6	54.9	71.4	66.9	67.4	18.4	77.1	78.0	38.2	38.9	2.5	57.2	61.0
BYOL [18]	RN-50	36.9	31.1	38.3	47.5	71.3	69.2	69.5	78.4	78.3	82.4	39.4	39.8	52.6	56.7	68.9
Barlow Twins [36]	RN-50	38.5	35.0	44.9	47.1	68.7	69.5	70.0	78.8	77.2	81.1	39.8	39.5	55.6	55.5	64.7
DenseCL [30]	RN-50	29.4	31.9	13.6	48.5	71.9	59.5	60.2	54.5	73.1	80.0	30.0	30.5	20.0	47.6	64.9
DINO [5]	RN-50	33.9	32.3	43.9	48.1	70.9	71.0	70.7	80.2	78.8	82.5	36.0	36.3	52.5	53.3	67.7
MoCo v3 [11]	RN-50	40.4	35.6	47.5	50.9	71.6	70.7	70.2	80.1	78.7	83.0	45.1	43.5	58.6	59.1	69.9
DINO [5]	ViT-B/16	39.5	40.3	56.7	57.3	71.5	78.8	79.0	81.1	81.9	86.0	48.4	48.7	58.7	59.1	71.0
iBOT [37]	ViT-B/16	41.6	42.5	60.1	60.4	72.7	79.0	79.2	81.3	82.4	87.0	48.2	48.2	59.5	60.1	73.9
MoCo v3 [11]	ViT-B/16	42.1	42.8	32.4	59.3	68.6	75.3	75.7	75.4	81.1	85.3	43.6	44.1	35.2	56.8	67.6
MAE [19]	ViT-B/16	40.9	44.7	28.8	57.4	70.7	35.2	61.0	60.4	76.2	83.2	22.9	34.2	20.3	50.4	66.1
MaskFeat [31]	ViT-B/16	22.6	31.3	2.0	42.5	67.2	25.3	40.8	4.2	61.4	78.2	12.9	18.5	2.5	32.8	55.7
BEiT v2 [29]	ViT-B/16	43.7	44.7	56.4	58.7	73.5	78.2	78.4	83.7	83.5	88.8	48.4	48.7	63.1	62.9	76.3
MILAN [20]	ViT-B/16	48.5	49.0	40.0	59.3	71.5	82.9	83.0	84.2	86.0	88.8	57.0	57.5	55.6	66.0	75.4
EVA [15]	ViT-B/16	49.4	50.8	34.4	58.4	70.9	61.0	64.8	64.7	77.9	85.6	30.0	32.4	21.9	51.1	67.0
PixMIM [23]	ViT-B/16	42.1	44.5	28.0	55.3	69.1	51.6	63.0	61.9	76.1	82.1	25.8	34.2	20.4	50.4	61.2

iNaturalist mini

		Target: Family					Target: Genus					Target: Species				
Method	Backbone	kNN	kNN^{N}	LP	LP^{BN}	FT	kNN	kNN^{N}	LP	LP^{BN}	FT	kNN	kNN^{N}	LP	LP^{BN}	FT
Jigsaw [26]	RN-50	11.4	11.6	11.9	19.6	72.7	2.2	2.2	4.1	6.3	42.7	1.4	1.6	2.8	3.9	34.7
rotnet [16]	RN-50	13.4	13.4	19.9	23.1	72.9	3.0	3.0	6.0	9.7	46.3	2.1	2.3	4.2	6.4	38.0
npid [33]	RN-50	21.0	21.0	17.6	31.3	71.5	8.0	8.2	5.5	17.2	42.1	5.5	5.8	3.6	12.1	36.5
Sela-v2 [1]	RN-50	31.7	33.2	39.6	42.7	76.5	14.1	16.4	21.1	29.9	60.5	9.7	10.7	14.3	20.5	48.6
npid++ [24, 33]	RN-50	20.8	21.2	31.0	31.3	75.5	7.8	8.1	15.6	17.4	54.1	5.5	5.6	11.2	12.2	45.2
PIRL [24]	RN-50	26.9	27.1	19.4	35.3	75.7	11.0	11.6	18.2	21.3	54.7	7.6	7.9	12.9	15.1	44.8
clusterfit [35]	RN-50	19.9	20.5	29.2	29.3	72.8	6.5	7.0	12.8	13.2	44.7	4.2	4.6	8.5	8.8	34.5
Deepcluster-v2 [4, 6]	RN-50	38.1	40.3	49.8	50.5	77.7	20.1	22.9	36.2	39.6	62.2	13.7	16.2	26.3	29.2	50.8
SwAV [6]	RN-50	36.0	38.6	47.0	48.4	77.7	17.1	21.2	30.2	36.2	61.6	12.2	14.8	21.3	26.1	49.9
SimCLR [8]	RN-50	29.1	29.4	34.0	40.4	75.2	13.4	13.9	15.9	26.2	55.0	8.8	9.7	10.5	17.5	44.0
MoCo v2 [12]	RN-50	32.6	33.6	28.4	48.1	77.3	16.5	17.3	9.3	34.7	55.1	10.8	11.8	6.2	25.3	46.5
SimSiam [10]	RN-50	32.8	33.6	9.5	45.9	75.6	15.9	17.7	8.4	33.0	45.5	10.7	12.3	5.0	24.2	40.3
BYOL [18]	RN-50	37.6	39.3	45.1	48.3	76.3	20.3	24.6	28.5	38.3	60.9	14.1	17.1	20.2	27.8	50.1
Barlow Twins [36]	RN-50	37.6	39.0	47.3	47.8	66.7	20.2	22.8	33.9	36.3	50.4	13.7	16.0	24.6	26.7	38.8
DenseCL [30]	RN-50	26.0	26.7	25.0	38.5	76.7	11.3	12.2	7.6	24.1	52.5	7.1	7.7	5.2	17.1	44.2
DINO [5]	RN-50	40.1	40.5	50.1	51.3	77.8	21.8	23.0	33.4	38.4	60.7	14.8	15.6	23.1	27.3	49.0
MoCo v3 [11]	RN-50	40.5	40.0	47.4	48.6	77.5	25.8	26.9	35.2	40.3	64.4	18.1	19.4	26.1	29.7	53.1
DINO [5]	ViT-B/16	55.8	56.4	60.6	61.1	82.2	44.5	45.3	60.5	61.5	71.3	32.6	33.0	49.0	48.9	59.1
iBOT [37]	ViT-B/16	53.4	54.1	60.6	61.2	84.1	42.0	43.3	60.6	61.1	73.9	31.5	32.1	47.9	48.5	61.7
MoCo v3 [11]	ViT-B/16	45.0	45.7	43.4	56.2	75.2	29.0	30.0	23.6	51.8	66.7	20.6	21.4	16.8	40.0	55.6
MAE [19]	ViT-B/16	13.7	25.7	30.8	44.5	79.6	3.0	10.2	7.8	31.9	65.3	2.4	7.2	5.0	23.5	54.5
MaskFeat [31]	ViT-B/16	10.0	17.9	8.2	31.4	75.2	2.0	5.1	0.5	15.1	46.4	1.5	3.6	0.5	10.3	39.0
BEiT v2 [29]	ViT-B/16	43.1	44.1	58.9	59.2	85.4	18.7	19.6	44.5	44.6	79.2	12.6	13.1	33.7	33.5	68.4
MILAN [20]	ViT-B/16	53.5	54.2	50.7	61.3	84.5	36.4	37.2	34.7	56.2	74.7	27.0	27.6	26.4	45.7	64.4
EVA [15]	ViT-B/16	27.6	30.8	34.8	49.4	82.0	8.8	11.2	8.6	30.9	69.9	6.3	8.2	5.8	22.0	59.3
PixMIM [23]	ViT-B/16	20.5	27.1	31.4	44.7	76.9	6.0	10.7	8.5	32.1	61.3	4.0	7.6	5.6	22.8	50.9

Table S.3: *Top-5* classification accuracies (in %). LP: Linear Probing, FT: End-to-end Fine-tuning, FT 10%/1%: Few-Shot Fine-tuning on 10%/1% of the training data, kNN: k-nearest neighbors probing. The superscript "BN" denotes the use of a final batch norm layer. The superscript "N" is the equivalent for k-nearest neighbors probing where the embedding is normalization on training split statistics.

		ImageNet									PascalVOC					Cifar100					Caltech-256					CUB				
Method	Backbone	kNN	kNN^{N}	LP	LP^{BN}	FT	FT10%	FT10%^{BN}	FT1%	FT1%^{BN}	kNN	kNN^{N}	LP	LP^{BN}	FT	kNN	kNN^{N}	LP	LP^{BN}	FT	kNN	kNN^{N}	LP	LP^{BN}	FT	kNN	kNN^{N}	LP	LP^{BN}	FT
Jigsaw [26]	RN-50	24.8	25.4	52.2	56.4	90.5	76.6	77.9	39.3	45.8	75.6	75.3	85.1	86.6	92.1	51.8	52.7	54.2	65.6	94.8	45.3	46.2	50.1	64.4	89.7	11.4	12.8	16.6	23.1	82.3
rotnet [16]	RN-50	34.8	34.3	59.8	65.4	90.7	78.0	78.8	42.8	48.3	79.4	80.3	87.9	88.8	92.4	66.7	66.1	65.7	74.3	95.4	55.8	55.9	66.0	76.7	89.6	16.1	16.1	23.5	33.1	86.5
npid [33]	RN-50	57.9	58.4	56.5	74.8	90.2	76.5	79.7	27.1	54.8	87.0	86.5	70.8	92.3	86.5	68.2	66.9	50.2	82.4	95.2	68.5	69.1	33.0	85.2	85.4	34.9	35.2	31.4	56.2	67.2
Sela-v2 [1]	RN-50	80.1	80.4	90.2	89.7	93.3	89.4	89.1	77.1	80.2	94.8	92.8	97.7	98.3	98.9	84.4	82.5	67.3	74.7	98.0	85.7	86.1	89.2	92.9	96.8	38.5	42.2	47.6	71.3	91.7
npid++ [24, 33]	RN-50	58.4	60.0	78.6	77.2	91.1	81.0	81.6	53.5	58.9	86.1	85.8	95.5	94.3	96.2	68.4	67.1	74.1	82.9	96.4	67.1	67.1	84.2	85.9	92.1	33.6	35.1	49.2	59.8	91.6
PIRL [24]	RN-50	71.4	71.8	81.9	81.8	91.8	83.6	83.6	61.9	66.4	91.8	90.1	95.5	94.5	97.2	75.4	71.8	65.3	77.6	96.5	78.7	79.4	87.7	88.8	93.9	38.5	37.4	48.6	58.6	91.5
clusterfit [35]	RN-50	56.2	57.0	72.8	74.0	90.5	78.7	79.7	48.5	55.4	86.5	83.0	92.4	92.2	93.6	77.2	77.0	87.2	87.4	96.0	65.5	71.4	85.1	85.3	91.8	19.9	24.7	42.5	44.7	84.9
Deepcluster-v2 [4, 6]	RN-50	83.8	84.2	92.0	91.2	93.4	89.8	89.9	76.3	81.3	96.2	96.1	98.5	98.2	98.6	86.2	86.3	74.0	76.7	98.2	89.2	89.4	94.5	95.6	97.2	53.2	61.5	68.1	82.7	93.2
SwAV [6]	RN-50	82.9	83.5	91.8	91.2	93.4	89.6	90.0	73.4	81.1	96.1	96.1	98.5	98.6	98.8	86.2	86.3	72.5	75.6	97.9	88.6	89.2	93.4	95.2	96.9	48.5	57.5	59.1	80.3	92.2
SimCLR [8]	RN-50	78.1	76.7	87.9	88.5	92.5	87.9	88.0	75.4	79.6	94.1	91.5	97.6	98.3	99.3	82.2	78.3	61.9	79.3	98.0	83.8	83.0	88.1	92.8	96.1	41.7	39.7	45.1	67.2	90.4
MoCo v2 [12]	RN-50	80.6	79.3	83.8	89.8	92.3	84.8	85.5	53.0	74.8	96.0	95.5	91.9	98.6	96.7	83.9	82.5	60.1	90.1	96.8	86.8	86.8	65.6	94.6	93.3	45.3	48.4	39.3	74.8	87.5
SimSiam [10]	RN-50	79.0	79.0	67.0	88.9	91.8	79.3	84.0	24.2	66.5	95.5	94.8	47.9	97.8	85.4	85.9	83.5	46.5	86.9	95.1	86.9	86.5	10.3	94.6	87.0	46.9	49.2	26.0	77.3	65.4
BYOL [18]	RN-50	82.1	82.3	90.9	90.5	93.0	89.1	89.0	76.4	80.7	95.8	95.1	98.5	98.1	99.0	87.0	84.3	82.1	86.0	98.1	87.8	87.3	93.6	95.4	96.5	62.7	67.2	71.0	84.3	93.5
Barlow Twins [36]	RN-50	81.1	81.2	89.9	89.4	91.9	87.4	86.5	73.9	76.3	95.9	95.2	98.0	97.6	98.3	85.5	82.9	83.5	83.4	97.7	88.4	88.1	93.8	94.6	95.7	58.4	64.0	71.4	82.2	86.8
DenseCL [30]	RN-50	72.5	72.8	76.6	85.6	92.0	83.7	84.6	54.6	69.8	95.7	96.2	96.1	97.2	97.6	76.4	77.6	56.9	84.1	96.8	83.4	83.5	76.8	91.2	93.3	35.4	38.1	31.3	61.3	88.6
DINO [5]	RN-50	83.8	83.4	92.1	91.3	93.3	89.4	89.8	72.9	80.6	96.2	96.0	98.5	98.2	99.0	82.8	81.4	74.8	78.3	98.0	89.5	88.8	93.7	95.2	97.0	57.1	62.0	60.7	80.4	92.6
MoCo v3 [11]	RN-50	84.2	83.8	91.8	91.0	93.4	89.7	90.0	77.7	82.5	96.5	95.0	98.4	97.9	98.7	87.5	82.9	85.4	86.9	98.2	89.5	88.0	95.3	95.5	96.8	73.0	70.4	78.4	86.4	94.4
DINO [5]	ViT-B/16	91.1	91.2	93.1	93.6	95.6	92.8	93.7	87.9	90.2	97.5	97.5	99.2	99.1	99.0	94.1	93.7	97.2	97.3	99.0	96.1	96.3	97.5	97.9	98.1	85.7	86.0	95.0	95.1	96.3
iBOT [37]	ViT-B/16	91.7	91.7	93.8	94.2	96.2	94.0	94.5	89.9	91.8	97.9	98.2	99.2	99.1	99.0	93.7	93.6	97.2	97.2	99.3	96.1	96.4	97.8	98.0	98.4	84.2	85.0	94.9	95.3	96.4
MoCo v3 [11]	ViT-B/16	88.3	88.7	91.9	93.0	95.5	92.5	92.5	60.2	88.0	96.8	96.5	97.5	99.3	98.2	93.5	93.7	95.1	97.1	98.9	92.8	93.3	93.5	97.2	96.3	72.6	74.0	72.8	92.1	86.9
MAE [19]	ViT-B/16	44.1	68.6	81.0	86.8	94.6	88.8	89.6	71.3	79.7	71.1	89.8	82.1	97.5	98.6	46.4	75.4	71.7	90.4	98.2	47.1	79.0	59.1	92.6	95.9	16.4	37.3	20.1	73.9	92.6
MaskFeat [31]	ViT-B/16	26.0	48.9	10.7	75.5	92.6	80.5	86.0	23.7	71.0	72.5	83.4	52.0	92.7	88.0	56.2	71.5	28.6	85.9	96.7	38.0	51.9	11.5	79.7	89.5	12.7	17.0	6.4	36.6	66.5
BEiT v2 [29]	ViT-B/16	86.9	87.2	94.3	94.1	97.4	95.8	95.9	90.5	92.4	97.6	97.6	99.3	99.2	99.8	92.3	92.2	97.2	97.2	99.3	92.6	92.2	97.5	97.6	99.1	41.7	43.2	78.0	80.5	97.1
MILAN [20]	ViT-B/16	89.9	90.8	94.2	94.9	97.4	95.5	95.5	90.8	91.8	98.1	98.2	99.5	99.5	99.9	90.3	90.5	92.6	96.0	99.0	96.3	96.4	97.1	98.5	99.2	85.0	85.0	84.6	94.4	96.5
EVA [15]	ViT-B/16	68.3	72.8	83.1	88.8	96.0	91.2	91.8	70.2	79.3	91.2	93.1	83.4	97.7	99.0	81.8	84.9	83.6	95.4	98.5	74.8	79.5	54.8	94.6	97.1	28.6	32.5	27.2	69.1	89.8
PixMIM [23]	ViT-B/16	59.4	70.5	81.7	87.0	94.7	86.2	87.6	52.0	71.5	84.9	90.6	83.8	98.0	97.7	71.1	83.0	78.7	93.2	97.2	67.3	80.4	59.1	92.9	94.3	25.7	35.8	23.4	70.9	80.0

		ImageNet-D																													
		Clipart					Infograph					Painting					Quickdraw					Real					Sketch				
Method	Backbone	kNN	kNN^{N}	LP	LP^{BN}	FT	kNN	kNN^{N}	LP	LP^{BN}	FT	kNN	kNN^{N}	LP	LP^{BN}	FT	kNN	kNN^{N}	LP	LP^{BN}	FT	kNN	kNN^{N}	LP	LP^{BN}	FT	kNN	kNN^{N}	LP	LP^{BN}	FT
Jigsaw [26]	RN-50	31.4	32.3	42.1	58.2	83.7	18.2	18.3	22.6	28.1	50.9	28.4	28.1	44.9	55.4	77.5	55.0	56.9	38.4	53.0	92.5	52.9	52.9	69.0	77.0	91.2	28.7	29.6	35.9	48.3	78.1
rotnet [16]	RN-50	42.0	42.9	56.2	68.3	85.9	18.2	19.8	27.8	33.6	54.2	38.1	37.5	57.4	64.6	79.3	59.9	59.9	62.0	76.8	92.7	60.8	60.8	76.0	81.8	92.0	38.0	38.7	50.4	61.1	80.2
npid [33]	RN-50	49.0	48.3	22.4	69.0	83.1	27.4	27.7	15.0	35.3	51.2	47.6	47.6	35.7	67.4	77.2	65.6	62.5	26.4	73.2	92.1	74.2	74.5	62.7	84.3	91.2	45.5	44.5	21.9	60.4	77.8
Sela-v2 [1]	RN-50	55.9	54.5	70.8	76.2	90.0	31.2	32.9	40.7	44.0	62.8	61.0	61.2	77.4	78.9	87.3	55.1	49.1	60.4	71.3	92.2	86.0	86.2	91.3	91.6	94.8	51.4	51.2	65.8	69.8	84.9
npid++ [24, 33]	RN-50	42.7	40.7	64.6	68.3	86.4	27.2	27.5	34.6	35.0	54.4	45.8	45.3	67.4	66.6	80.1	48.4	37.8	56.8	71.6	92.5	74.3	74.3	86.8	85.5	92.3	35.8	35.3	51.2	55.8	80.5
PIRL [24]	RN-50	56.2	55.1	70.9	76.5	87.5	30.3	32.1	38.0	41.8	57.0	56.9	56.6	72.5	74.0	82.0	57.1	45.2	59.5	68.2	92.5	81.7	81.9	88.3	88.7	92.8	52.4	51.3	63.6	68.4	81.8
clusterfit [35]	RN-50	55.2	56.9	72.6	73.4	85.5	27.2	28.8	36.6	37.0	52.8	53.3	54.1	68.6	69.7	79.7	66.7	67.0	78.5	78.9	91.5	76.8	77.3	85.5	85.9	91.9	50.0	51.0	64.5	65.1	78.9
Deepcluster-v2 [4, 6]	RN-50	61.5	62.2	79.9	80.3	90.5	35.3	37.1	47.8	47.4	62.4	64.7	65.3	83.0	82.1	87.7	57.5	58.0	72.1	75.8	92.4	88.5	88.7	93.6	92.7	94.9	56.1	56.5	73.3	73.3	85.5
SwAV [6]	RN-50	59.7	60.7	78.4	79.5	90.2	35.1	36.9	46.5	47.1	62.7	64.4	65.0	82.1	81.6	87.5	55.9	54.1	73.3	78.2	92.4	88.2	88.5	93.2	92.3	94.9	55.0	55.9	72.0	73.3	85.6
SimCLR [8]	RN-50	54.6	54.8	61.9	77.2	89.8	30.6	33.3	32.3	42.3	60.8	60.4	59.8	72.3	78.5	86.2	49.5	35.4	51.9	75.0	92.5	84.5	84.3	88.6	90.8	94.2	51.9	52.1	58.1	71.2	84.2
MoCo v2 [12]	RN-50	58.1	60.7	33.0	81.8	87.6	31.9	35.2	18.1	48.3	57.1	64.3	64.2	53.4	81.0	82.5	57.0	53.6	35.7	76.0	92.7	86.1	86.3	80.8	92.3	93.2	55.3	56.7	31.8	75.7	81.9
SimSiam [10]	RN-50	60.5	62.2	5.3	82.6	84.5	33.3	36.3	6.3	48.5	52.4	65.1	64.9	7.3	82.0	78.7	63.5	59.8	32.4	81.8	92.5	86.7	86.8	31.0	92.3	92.0	58.5	59.2	6.0	76.7	78.5
BYOL [18]	RN-50	61.2	62.6	78.6	82.7	90.9	33.8	36.7	43.4	47.4	62.7	65.6	65.0	81.7	82.3	87.3	61.2	54.3	66.1	75.9	92.6	87.4	87.4	92.8	92.6	94.7	59.2	59.6	73.3	76.2	85.5
Barlow Twins [36]	RN-50	61.1	62.2	82.0	81.8	88.1	34.5	36.3	47.3	46.6	56.3	65.4	64.9	82.3	80.9	85.0	62.9	59.2	72.5	75.0	91.0	87.7	87.8	93.0	91.9	94.0	59.6	59.0	75.4	75.0	83.0
DenseCL [30]	RN-50	51.6	52.5	41.0	76.0	87.4	28.6	29.8	23.6	42.0	56.6	59.0	58.9	56.4	75.8	82.4	52.8	55.7	33.5	76.4	92.7	82.1	82.6	79.0	90.2	93.0	50.1	50.5	39.1	68.9	81.6
DINO [5]	RN-50	59.8	60.3	80.5	81.0	90.1	35.3	37.4	48.1	47.6	62.7	65.0	64.6	82.9	82.2	87.3	57.9	55.7	72.1	76.6	92.2	88.5	88.4	93.6	92.8	94.9	56.1	56.4	73.4	73.8	85.2
MoCo v3 [11]	RN-50	68.0	66.6	83.7	84.0	91.3	35.1	36.7	48.1	48.5	63.7	68.1	66.7	84.1	83.4	88.1	65.6	59.9	74.8	78.6	92.8	88.3	87.8	93.6	93.0	95.0	64.4	63.1	78.2	78.1	86.1
DINO [5]	ViT-B/16	73.4	73.5	85.0	85.6	92.1	47.2	47.0	56.5	57.5	65.0	75.7	75.5	85.0	85.6	89.1	64.5	65.4	82.8	83.2	92.5	93.0	92.9	94.1	94.5	95.8	68.2	68.3	77.6	78.1	86.9
iBOT [37]	ViT-B/16	74.6	74.4	86.5	87.0	93.4	47.8	47.1	57.4	58.5	67.1	76.1	75.8	85.8	86.5	90.7	66.7	67.6	85.2	85.5	93.1	93.1	93.1	94.5	94.8	96.3	67.6	67.1	78.0	78.7	88.6
MoCo v3 [11]	ViT-B/16	71.1	71.5	60.9	85.3	89.5	42.7	42.9	35.9	54.2	60.6	71.8	72.3	73.8	84.3	88.7	67.4	68.2	58.7	85.0	91.1	91.3	91.4	91.4	94.1	95.9	63.8	64.3	56.1	76.2	84.8
MAE [19]	ViT-B/16	39.2	62.9	47.3	81.0	89.7	21.0	33.7	25.2	47.4	61.2	28.7	56.4	54.0	78.2	87.0	66.6	70.8	55.5	83.1	92.2	58.9	82.0	81.9	91.4	94.8	40.8	54.8	39.2	71.2	83.8
MaskFeat [31]	ViT-B/16	29.4	37.5	8.1	65.7	81.3	16.1	21.3	8.0	32.3	46.5	24.1	39.2	11.1	66.3	76.7	44.0	55.3	7.7	70.6	89.7	46.7	63.8	14.8	82.3	90.6	26.1	34.7	7.9	54.3	74.6
BEiT v2 [29]	ViT-B/16	77.4	77.1	89.3	88.8	95.1	43.0	42.6	59.8	59.0	71.8	73.7	73.4	87.6	87.0	93.3	69.9	71.0	83.1	84.7	93.5	92.2	92.0	95.2	95.2	97.3	68.8	68.7	82.1	81.7	90.6
MILAN [20]	ViT-B/16	84.8	85.0	85.1	90.8	94.8	54.6	54.9	55.1	65.3	73.4	77.7	77.8	85.6	88.9	93.3	74.5	75.0	67.8	85.1	92.6	94.6	94.6	95.8	96.3	97.4	76.5	76.9	76.6	84.1	90.1
EVA [15]	ViT-B/16	58.5	62.1	50.3	82.4	90.4	34.6	36.3	28.6	51.0	64.3	55.3	59.1	57.9	79.8	88.8	75.5	76.5	62.2	84.6	92.2	82.8	85.3	85.7	92.8	96.0	50.0	52.9	41.4	72.7	84.9
PixMIM [23]	ViT-B/16	50.8	63.6	47.7	81.2	86.3	28.2	34.7	25.9	48.2	57.4	45.0	59.0	56.4	78.6	84.7	67.6	70.3	54.4	82.0	91.2	74.7	83.5	83.1	91.5	94.6	44.8	54.6	39.2	71.2	80.5

		iNaturalist mini														
		Target: Family					Target: Genus					Target: Species				
Method	Backbone	kNN	kNN^{N}	LP	LP^{BN}	FT	kNN	kNN^{N}	LP	LP^{BN}	FT	kNN	kNN^{N}	LP	LP^{BN}	FT
Jigsaw [26]	RN-50	25.0	25.4	28.7	41.4	89.5	6.3	6.9	12.4	17.1	66.7	4.7	5.1	9.4	12.4	62.3
rotnet [16]	RN-50	28.1	28.3	42.0	46.6	89.6	8.2	8.2	16.5	22.7	69.8	6.5	6.6	13.6	18.0	65.9
npid [33]	RN-50	40.2	40.4	37.5	56.6	88.8	17.6	18.4	14.9	34.6	66.7	14.1	14.4	12.6	28.3	64.0
Sela-v2 [1]	RN-50	55.6	57.1	66.2	69.2	91.7	30.0	33.4	42.1	52.0	81.3	23.2	25.9	34.5	43.5	76.9
npid++ [24, 33]	RN-50	41.0	41.2	57.7	57.8	90.7	18.1	19.0	34.4	36.8	75.1	13.9	14.4	27.8	29.9	71.0
PIRL [24]	RN-50	48.8	48.6	39.5	61.6	90.8	24.3	25.0	38.4	42.1	76.1	18.4	19.1	31.3	34.4	71.6
clusterfit [35]	RN-50	38.5	39.0	54.2	54.4	89.2	14.9	15.9	28.0	28.8	67.2	11.1	11.7	21.4	22.2	61.4
Deepcluster-v2 [4, 6]	RN-50	62.3	64.2	74.7	75.0	92.2	37.2	41.4	58.8	61.8	82.0	29.7	33.6	51.2	54.1	77.5
SwAV [6]	RN-50	60.0	62.5	72.9	73.8	92.2	33.9	39.3	52.4	58.2	81.6	26.3	31.1	44.5	50.4	77.0
SimCLR [8]	RN-50	52.1	51.8	59.9	67.0	90.8	27.7	29.2	34.0	47.4	78.1	21.2	22.3	27.1	38.8	72.8
MoCo v2 [12]	RN-50	56.7	57.4	52.1	73.7	91.9	33.5	34.7	25.7	58.6	77.4	26.0	27.7	20.3	50.1	73.9
SimSiam [10]	RN-50	55.9	56.5	20.8	71.6	91.0	32.1	34.4	20.5	55.9	69.5	24.8	26.8	15.8	48.1	67.9
BYOL [18]	RN-50	60.8	62.4	70.6	73.3	91.6	38.5	43.8	50.9	61.3	81.5	30.5	35.7	43.0	52.9	77.1
Barlow Twins [36]	RN-50	60.8	62.0	72.9	72.9	87.1	38.0	41.4	56.3	58.4	73.2	29.8	33.8	48.9	51.1	65.7
DenseCL [30]	RN-50	48.8	49.5	49.2	65.5	91.5	25.5	26.9	21.0	46.6	75.2	19.0	19.8	16.4	38.4	72.7
DINO [5]	RN-50	63.8	64.0	75.4	76.2	92.2	40.0	41.3	55.7	61.1	81.4	31.5	33.0	47.2	52.3	76.5
MoCo v3 [11]	RN-50	63.8	62.7	72.5	73.1	92.0	44.8	46.3	58.3	62.7	82.6	37.3	39.4	51.2	55.2	78.7
DINO [5]	ViT-B/16	78.8	79.1	83.2	83.7	94.3	68.5	68.6	80.0	81.2	87.0	59.7	60.1	74.3	75.8	83.3
iBOT [37]	ViT-B/16	77.5	77.9	83.5	84.0	95.1	66.6	66.9	80.4	81.5	88.5	58.3	58.5	74.2	75.8	85.3
MoCo v3 [11]	ViT-B/16	70.3	70.9	69.5	80.8	92.2	52.0	53.0	48.4	74.6	85.4	42.4	43.9	41.2	67.2	81.4
MAE [19]	ViT-B/16	29.4	47.3	55.7	71.3	93.3	8.4	22.9	20.1	55.8	84.3	6.6	17.9	16.4	48.3	80.2
MaskFeat [31]	ViT-B/16	22.1	35.2	20.7	57.4	90.0	5.5	12.8	2.2	32.3	70.5	4.7	9.7	2.0	25.4	67.5
BEiT v2 [29]	ViT-B/16	67.3	68.2	83.5	83.8	96.3	36.2	37.1	69.3	69.1	91.8	27.2	27.9	61.1	60.8	89.3
MILAN [20]	ViT-B/16	78.2	78.6	78.3	85.5	96.0	60.7	61.4	62.1	79.0	90.5	51.7	52.3	55.3	73.3	87.1
EVA [15]	ViT-B/16	50.4	54.4	60.8	76.2	95.0	20.9	24.4	22.1	54.6	87.5	15.4	18.2	17.8	45.8	83.8
PixMIM [23]	ViT-B/16	40.1	49.6	56.8	71.5	92.6	15.4	23.7	21.9	55.4	82.0	11.0	17.9	18.1	47.1	78.3

C Additional Visualizations

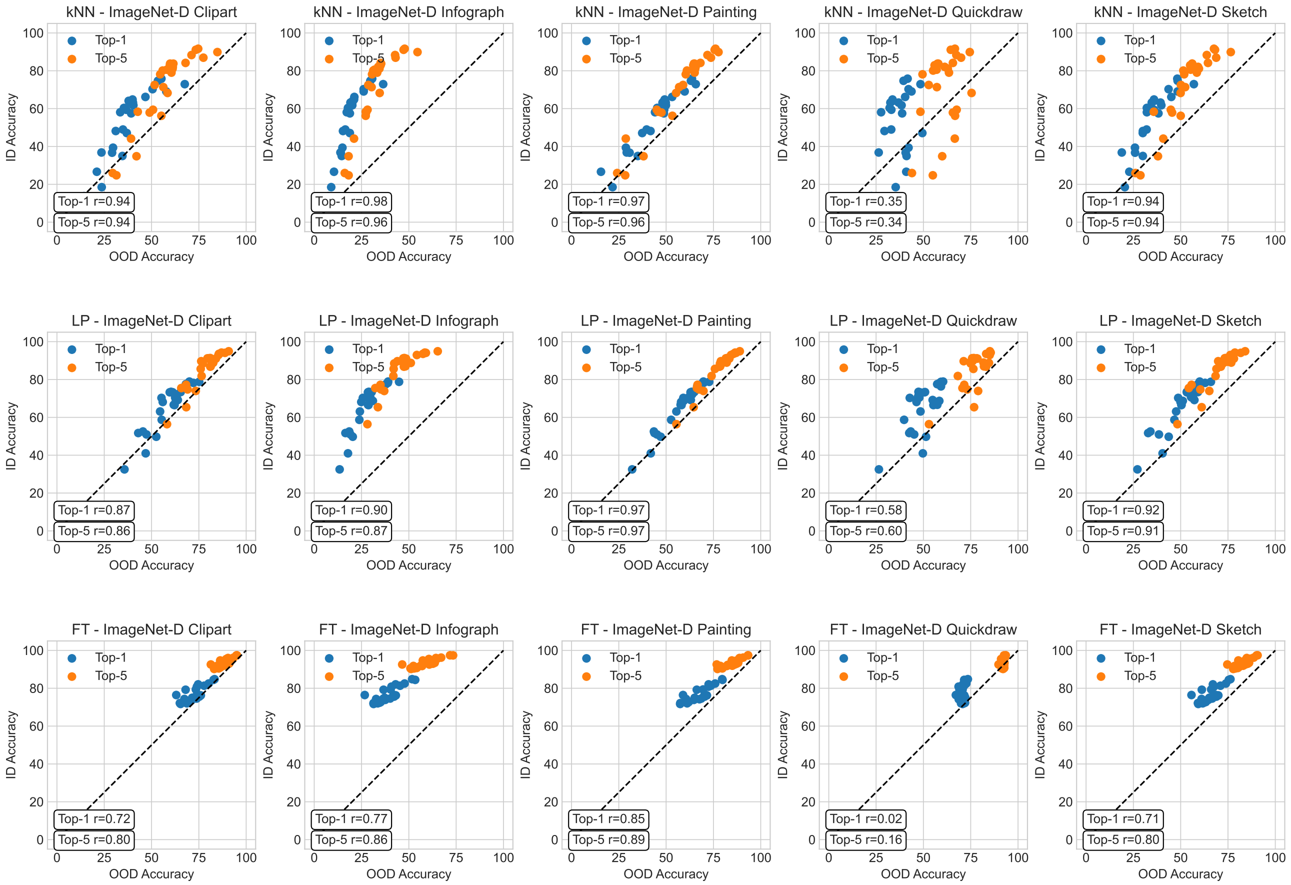

Figure S.1: ImageNet-D ID vs. OOD accuracies on different protocols. We compare both top-1 and top-5 classification accuracies. Correlation coefficients r are calculated using Spearman's rank correlation.

D Fine-grained experiments

The iNaturalist data set comes with different levels of hierarchical classes. Taxonomic closeness is a rough proxy for the degree of visual similarity and implies different degrees of fine-graininess of visual features [13]. We use the three most fine-grained targets, "Family", "Genus", and "Species" to estimate whether evaluation protocols or models are more or less sensitive to fine-grained features than others. For "Family" we use the full iNat mini data set comprising 1103 classes. By definition, more fine-grained classes would increase the number of classes when the whole dataset is used. To ensure a fair comparison with "Genus" and "Species" targets, we create subsampled datasets that have the same number of classes. At the same time, we ensure that the number of higher-level categories for a subset is as small as possible. In detail, we realize this as follows: For the "Genus" subset, we select the 256 families from the "Chordata" phylum that contain the most species, resulting in 1103 "Genus" categories. For the "Species" subset, we sort the previously defined Genus subset by the number of species and pick the top 277 categories, resulting in 1103 "Species" targets.

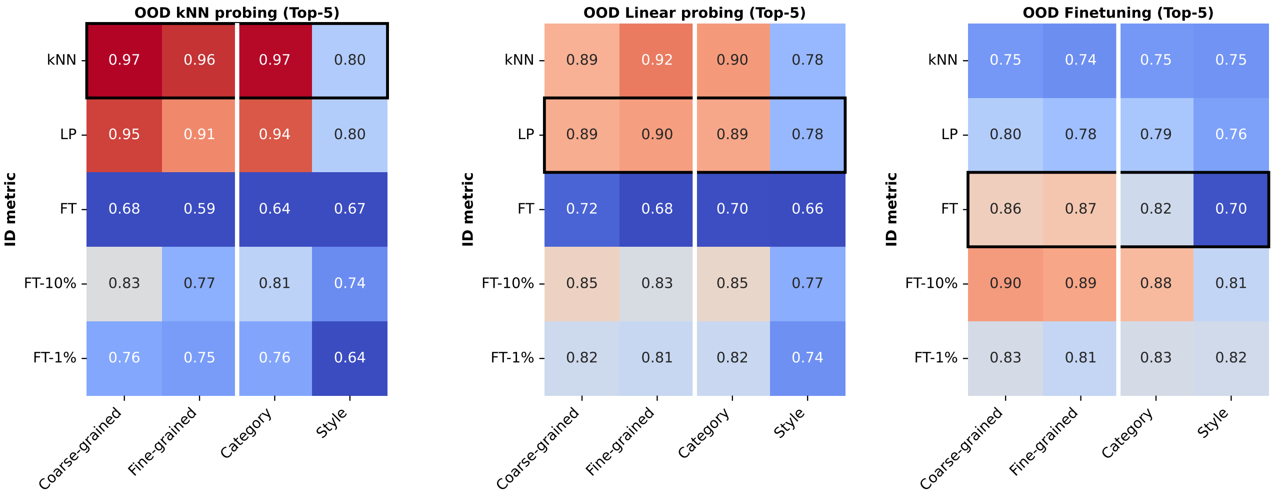

Figure S.2: Spearman rank correlations of *top-5* classification accuracies derived from in-domain and out-of-domain protocols under certain types of domain shift. See Fig. 3 (main paper) for more details.

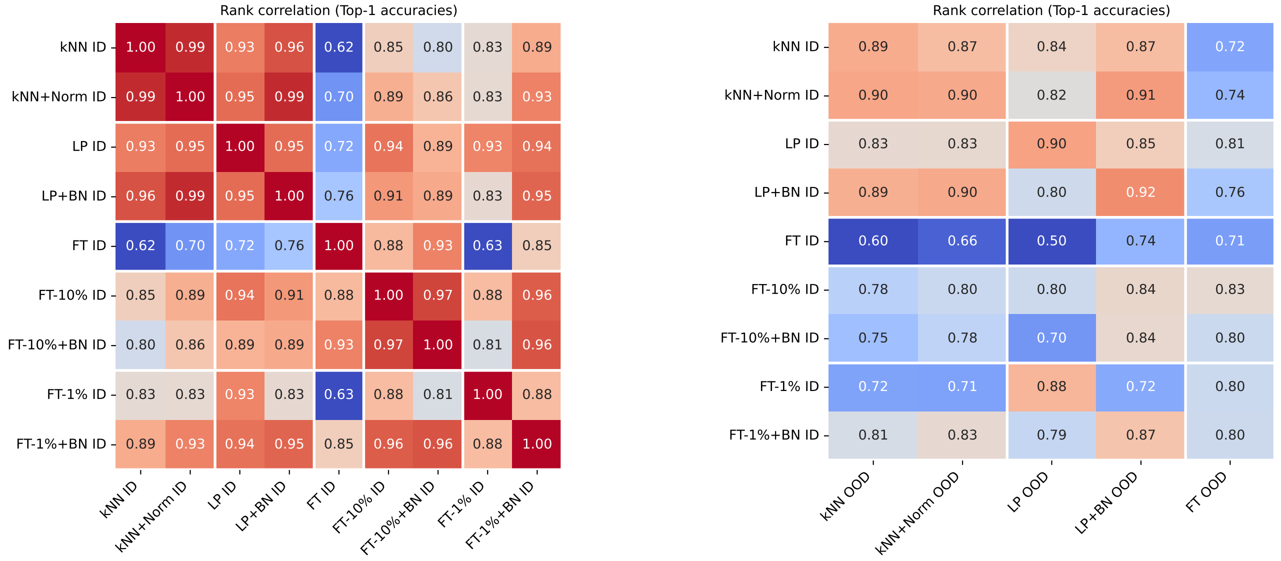

Figure S.3: Extended version of Fig. 2 (main paper). We show both versions, with and without feature normalization for all protocols but 100% fine-tuning.

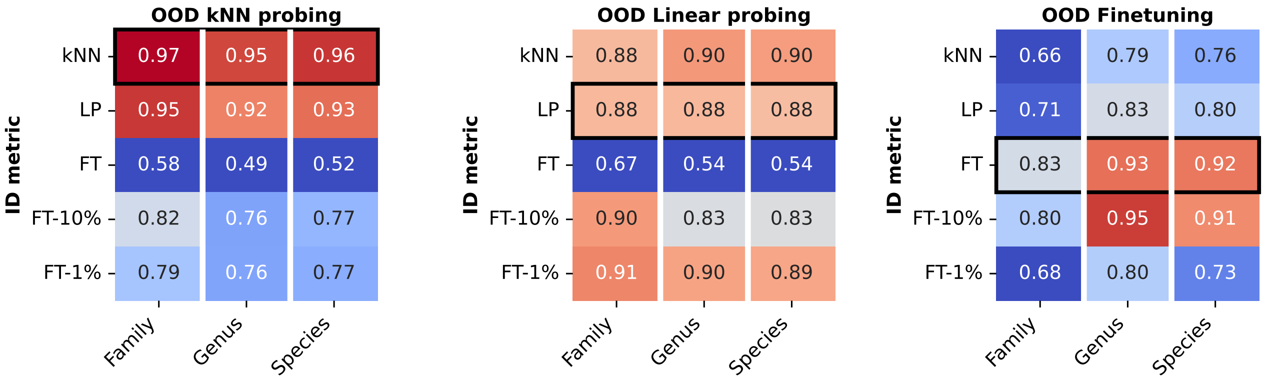

Figure S.4: Spearman rank correlations of top-1 classification accuracies for three different targets in the iNaturalist mini dataset.

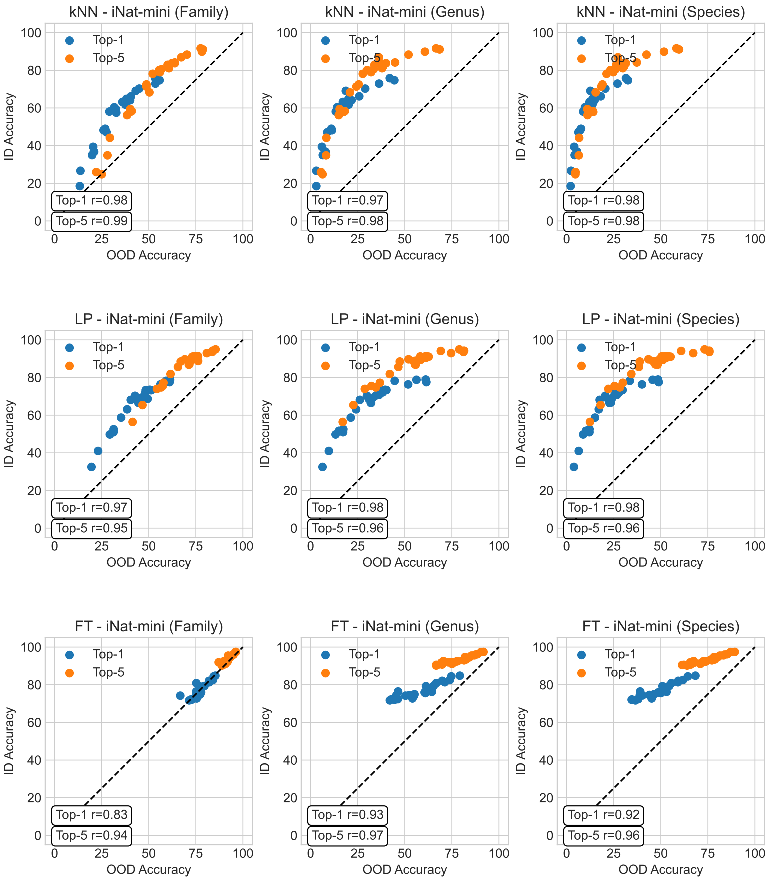

Figure S.5: iNaturalist ID vs. OOD accuracies with different targets (family, genus, species) on different protocols. We compare both top-1 and top-5 classification accuracies. Correlation coefficients r are calculated using Spearman's rank correlation.

E Implementation Details

All experiments were run using models implemented in PyTorch [27] using the `timm` [32] and `mmselfsup` [25] libraries. All end-to-end fine-tuning and linear probing were trained using a Stochastic Gradient Descent optimizer with 100 total epochs (5 warmup epochs followed by Cosine Annealing decay) and a base learning rate of 0.1. Few-shot fine-tuning on ImageNet was trained on 30 epochs only, as proposed by Grill et al. [18]. We found this few-shot setup to be the most stable compared to others that have different learning rates for head and backbone [see, e.g., 6, 36]. The effective batch sizes varied per data set and can be found in Table S.4. When training vision transformers, we also use layerwise learning rate decay (0.65), label smoothing (0.1), and drop path (0.2), inspired by He et al. [19].

Table S.4: Chosen effective batch sizes for different data sets used in this study.

Dataset	Fine-tuning	Linear Probing
ImageNet-1k (full)	256	1024
ImageNet-1k (10%)	256	-
ImageNet-1k (1%)	256	-
Pascal VOC	32	32
Caltech-256	32	32
CUB	32	32
Cifar100	128	128
iNaturalist mini, Family	256	1024
iNaturalist mini, Genus	256	1024
iNaturalist mini, Species	256	1024
ImageNet-D Clipart	128	128
ImageNet-D Infograph	128	128
ImageNet-D Painting	128	128
ImageNet-D Quickdraw	256	256
ImageNet-D Real	256	256
ImageNet-D Sketch	128	128

KNN-probing was implemented with `scikit-learn` [28] using Euclidean distance, a brute force solver, and $k = 20$ neighbors as proposed by Caron et al. [5].

For ResNet-50, we use the last 2048-dimensional (pre-logit) representation to report probing accuracy. Kolesnikov et al. [21] described that this consistently works better for this architecture than using any intermediate latent representation. For Vision Transformers, we follow the protocol of most implementations by training the linear classifier on the 768-dimensional `cls`-token.

Certain models—especially those using masked image modeling—achieve a significantly better linear probe accuracy when batch normalization is used between the model outputs and the linear classifier, as suggested by He et al. [19] and verified by Lee et al. [22]. Based on our own experiments, we can see that normalization has either a strong positive or an insignificant effect on the accuracy depending on the model (see Table S.2). Accordingly, we decided to use batch normalization for linear probing and also to normalize latent representations before kNN probing. Table S.5 lists the sources of the pre-trained model checkpoints we used.

Table S.5: Sources of the pre-trained model checkpoints used in this study.

Method	Backbone	Source
Jigsaw	RN-50	`VISSL model zoo`
rotnet	RN-50	`VISSL model zoo`
npid	RN-50	`VISSL model zoo`
Sela-v2	RN-50	`SwAV official Github`
npid++	RN-50	`VISSL model zoo`
PIRL	RN-50	`VISSL model zoo`
clusterfit	RN-50	`VISSL model zoo`
Deepcluster-v2	RN-50	`Deepcluster official Github`
SwAV	RN-50	`SwAV official Github`
SimCLR	RN-50	`VISSL model zoo`
MoCo v2	RN-50	`MoCoV2 official Github`
SimSiam	RN-50	`MMSelfSup model zoo`
BYOL	RN-50	`Github (inofficial)`
Barlow Twins	RN-50	`MMSelfSup model zoo`
DenseCL	RN-50	`DenseCL official Github`
DINO	RN-50	`DINO official Github`
MoCo v3	RN-50	`MoCoV3 official Github`
DINO	ViT-B/16	`DINO official Github`
iBOT	ViT-B/16	`iBOT official Github`
MoCo v3	ViT-B/16	`MoCoV3 official Github`
MAE	ViT-B/16	`MAE official Github`
MaskFeat	ViT-B/16	`MMSelfSup model zoo`
BEiT v2	ViT-B/16	`BEiT official Github`
MILAN	ViT-B/16	`MILAN official Github`
EVA	ViT-B/16	`MMSelfSup model zoo`
PixMIM	ViT-B/16	`MMSelfSup model zoo`

Table S.6: Overview of how datasets were categorized in our domain shift experiments (Fig. 3 main paper). We define domain shifts w.r.t. ImageNet-1k.

Dataset	Coarse	Fine	Category	Style
Pascal VOC	✓			
Caltech256	✓		✓	
CUB		✓	✓	
iNat mini Family	✓		✓	
iNat mini Genus		✓		
iNat mini Species		✓		
ImageNet-D Clipart				✓
ImageNet-D Infograph				✓
ImageNet-D Painting				✓
ImageNet-D Quickdraw				✓
ImageNet-D Sketch				✓

F Approximating Errors

Running the full set of experiments multiple times is costly. Therefore, we randomly selected one model per metric per dataset and ran the same experiment three times to approximate a representative error value (see Table S.7). We can see that errors are generally small and conclude that our metrics generated by a single run can be trusted.

Table S.7: Top-1 accuracies for three different seeds of different models on different datasets. Generally, metrics generated by evaluation protocols are highly reproducible. Note that we do not cover the uncertainty introduced by different pre-training setups.

Protocol	Dataset	Method	Backbone	Run 1	Run 2	Run 3	mean	std
Linear Probing	ImageNet	SimCLR	RN-50	66.87	66.82	66.85	66.85	0.02
Fine-tuning	ImageNet	DINO	RN-50	76.00	76.01	76.27	76.09	0.12
10% Fine-tuning	ImageNet	MILAN	ViT-B/16	78.92	78.91	78.91	78.91	0.01
1% Fine-tuning	ImageNet	EVA	ViT-B/16	41.10	46.65	46.65	44.80	2.62
Linear Probe	Pascal VOC	Deepcluster v2	RN-50	85.66	85.72	85.86	85.75	0.08
Fine-tuning	Pascal VOC	Jigsaw	RN-50	64.62	64.73	63.22	64.19	0.69
Linear Probe	Caltech-256	BEiT v2	ViT-B/16	90.22	90.25	90.26	90.24	0.02
Fine-tuning	Caltech-256	MoCo v3	RN-50	88.52	88.53	88.40	88.48	0.06
Linear Probe	CUB	DINO	ViT-B/16	78.17	78.41	78.48	78.35	0.13
Fine-tuning	CUB	SeLa v2	RN-50	68.23	68.93	68.16	68.44	0.35
Linear Probe	iNat mini (family)	SwAV	RN-50	46.94	47.27	47.13	47.11	0.14
Fine-tuning	iNat mini (family)	Barlowtwins	RN-50	66.72	66.71	66.86	66.76	0.07

G Computational Costs of Protocols

Table S.8: Estimated computational cost of different protocols. GPU-based metrics were estimated based on training on 2x NVIDIA RTX A5000. For k-NN, we neglect CPU-based time required by the classifier and only account for GPU time used for model inference. The values shown are for ImageNet-1k (in-domain) protocols. Hyperparameters for other datasets can be found in Section E.

			GPU Hours	
Protocol	Batch Size	Epochs	ResNet-50	ViT-B/16
kNN	1024	-	0.6	1.3
LP	1024	100	36	70
FT	256	100	94	184
10%-FT	128	30	3.5	6.5
1%-FT	128	30	1.0	1.5

References

[1] Yuki Markus Asano, Christian Rupprecht, and Andrea Vedaldi. “Self-labelling via simultaneous clustering and representation learning”. *International Conference on Learning Representations*. 2020.

[2] Alexei Baevski et al. "data2vec: A General Framework for Self-supervised Learning in Speech, Vision and Language". *International Conference on Machine Learning (ICML)*. 2022.

[3] Hangbo Bao, Li Dong, and Furu Wei. "BEiT: BERT Pre-Training of Image Transformers". *arXiv preprint arXiv:2106.08254* (2021).

[4] Mathilde Caron et al. "Deep Clustering for Unsupervised Learning of Visual Features". *European Conference on Computer Vision (ECCV)*. 2018.

[5] Mathilde Caron et al. "Emerging Properties in Self-Supervised Vision Transformers". *Proceedings of the IEEE/CVF International Conference on Computer Vision (ICCV)*. 2021, pp. 9650–9660.

[6] Mathilde Caron et al. "Unsupervised Learning of Visual Features by Contrasting Cluster Assignments". *Advances in Neural Information Processing Systems* 33 (2020), pp. 9912–9924.

[7] Mark Chen et al. "Generative Pretraining From Pixels". *Proceedings of the 37th International Conference on Machine Learning*. Vol. 119. PMLR, 2020, pp. 1691–1703.

[8] Ting Chen et al. "A Simple Framework for Contrastive Learning of Visual Representations". *Proceedings of the 37th International Conference on Machine Learning*. Vol. 119. Proceedings of Machine Learning Research. PMLR, 2020, pp. 1597–1607.

[9] Xiaokang Chen et al. "Context Autoencoder for Self-Supervised Representation Learning". *International Journal of Computer Vision* 132.1 (2024), pp. 208–223.

[10] Xinlei Chen and Kaiming He. "Exploring Simple Siamese Representation Learning". *2021 IEEE/CVF Conference on Computer Vision and Pattern Recognition (CVPR)* (2020), pp. 15745–15753.

[11] Xinlei Chen, Saining Xie, and Kaiming He. "An Empirical Study of Training Self-Supervised Vision Transformers". *2021 IEEE/CVF International Conference on Computer Vision (ICCV)*. 2021, pp. 9620–9629.

[12] Xinlei Chen et al. "Improved Baselines with Momentum Contrastive Learning". *arXiv preprint arXiv:2003.04297* (2020).

[13] Elijah Cole et al. "When does contrastive visual representation learning work?" *Proceedings of the IEEE/CVF Conference on Computer Vision and Pattern Recognition (CVPR)*. 2022, pp. 14755–14764.

[14] Xiaoyi Dong et al. "PeCo: Perceptual Codebook for BERT Pre-training of Vision Transformers". *Proceedings of the AAAI Conference on Artificial Intelligence*. Vol. 37. 2023, pp. 552–560.

[15] Yuxin Fang et al. "EVA: Exploring the Limits of Masked Visual Representation Learning at Scale". *2023 IEEE/CVF Conference on Computer Vision and Pattern Recognition (CVPR)* (2022), pp. 19358–19369.

[16] Spyros Gidaris, Praveer Singh, and Nikos Komodakis. "Unsupervised Representation Learning by Predicting Image Rotations". *International Conference on Learning Representations*. 2018.

[17] Priya Goyal et al. "Self-supervised Pretraining of Visual Features in the Wild". *arXiv preprint arXiv:2103.01988* (2021).

[18] Jean-Bastien Grill et al. "Bootstrap Your Own Latent a New Approach to Self-Supervised Learning". *Proceedings of the 34th International Conference on Neural Information Processing Systems*. 2020.

[19] Kaiming He et al. "Masked Autoencoders Are Scalable Vision Learners". *2022 IEEE/CVF Conference on Computer Vision and Pattern Recognition (CVPR)* (2021), pp. 15979–15988.

[20] Zejiang Hou et al. "MILAN: Masked image pretraining on language assisted representation". *arXiv preprint arXiv:2208.06049* (2022).

[21] Alexander Kolesnikov, Xiaohua Zhai, and Lucas Beyer. "Revisiting Self-Supervised Visual Representation Learning". *2019 IEEE/CVF Conference on Computer Vision and Pattern Recognition (CVPR)* (2019), pp. 1920–1929.

[22] Jae-Hun Lee et al. "Rethinking evaluation protocols of visual representations learned via self-supervised learning". *arXiv preprint arXiv:2304.03456* (2023).

[23] Yuan Liu et al. "Pixmim: Rethinking pixel reconstruction in masked image modeling". *arXiv preprint arXiv:2303.02416* (2023).

[24] Ishan Misra and Laurens van der Maaten. "Self-Supervised Learning of Pretext-Invariant Representations". *2020 IEEE/CVF Conference on Computer Vision and Pattern Recognition (CVPR)* (2019), pp. 6706–6716.

[25] MMSelfSup Contributors. *MMSelfSup: OpenMMLab Self-Supervised Learning Toolbox and Benchmark.* 2021. URL: `https://github.com/open-mmlab/mmselfsup`.

[26] Mehdi Noroozi and Paolo Favaro. "Unsupervised Learning of Visual Representations by Solving Jigsaw Puzzles". *European Conference on Computer Vision (ECCV)*. 2016.

[27] Adam Paszke et al. "PyTorch: An Imperative Style, High-Performance Deep Learning Library". *Advances in Neural Information Processing Systems (NeurIPS)* 32 (2019), pp. 8026–8037.

[28] Fabian Pedregosa et al. "Scikit-learn: Machine learning in Python". *Journal of Machine Learning Research* 12 (2011), pp. 2825–2830.

[29] Zhiliang Peng et al. "BEiT v2: Masked Image Modeling with Vector-Quantized Visual Tokenizers". *arXiv preprint arXiv:2208.06366* (2022).

[30] Xinlong Wang et al. "Dense Contrastive Learning for Self-Supervised Visual Pre-Training". *2021 IEEE/CVF Conference on Computer Vision and Pattern Recognition (CVPR)* (2020), pp. 3023–3032.

[31] Chen Wei et al. "Masked Feature Prediction for Self-Supervised Visual Pre-Training". *2022 IEEE/CVF Conference on Computer Vision and Pattern Recognition (CVPR)*. 2022, pp. 14648–14658. DOI: `10.1109/CVPR52688.2022.01426`.

[32] Ross Wightman. *PyTorch Image Models*. 2019. URL: `https://github.com/rwightman/pytorch-image-models`.

[33] Zhirong Wu et al. "Unsupervised Feature Learning via Non-parametric Instance Discrimination". *2018 IEEE/CVF Conference on Computer Vision and Pattern Recognition* (2018), pp. 3733–3742.

[34] Zhenda Xie et al. "SimMIM: A Simple Framework for Masked Image Modeling". *2022 IEEE/CVF Conference on Computer Vision and Pattern Recognition (CVPR)* (2021).

[35] Xueting Yan et al. "ClusterFit: Improving Generalization of Visual Representations". *2020 IEEE/CVF Conference on Computer Vision and Pattern Recognition (CVPR)* (2019), pp. 6508–6517.

[36] Jure Zbontar et al. "Barlow Twins: Self-Supervised Learning via Redundancy Reduction". *International Conference on Machine Learning*. 2021.

[37] Jinghao Zhou et al. "iBOT: Image BERT Pre-Training with Online Tokenizer". *arXiv preprint arXiv:2111.07832* (2021).
